# Bibliometric Study of Scientific Productivity on the Impacts on Mental Health in Times of Pandemic

**DOI:** 10.3390/medicina58010024

**Published:** 2021-12-24

**Authors:** Luz Marina Caballero-Apaza, Rubén Vidal-Espinoza, Silvia Curaca-Arroyo, Rossana Gomez-Campos, Zaida Callata-Gallegos, José Fuentes-López, Marco Cossio-Bolaños

**Affiliations:** 1 Escuela Profesional de Enfermería, Universidad Nacional del Altiplano de Puno, Puno 21001, Peru; lmcaballero@unap.edu.pe (L.M.C.-A.); sdcuraca@unap.edu.pe (S.C.-A.); 2 Instituto de Investigación en Ciencias de la Educación (IICE), Universidad Nacional del Altiplano de Puno, Puno 21001, Peru; zaidacallata@unap.edu.pe (Z.C.-G.); jdfuentes@unap.edu.pe (J.F.-L.); 3 Facultad de Educación, Universidad Católica Silva Henriquez, Santiago 8330225, Chile; rvidal@gmail.com; 4 Departamento de Diversidad e Inclusividad Educativa, Universidad Católica del Maule, Talca 3466706, Chile; rossaunicamp@gmail.com; 5 Departamento de Ciencias de la Actividad Física, Universidad Católica del Maule, Talca 3466706, Chile

**Keywords:** mental health, COVID-19, bibliometrics

## Abstract

*Background and Objectives:* The presence of the new SARS-CoV-2 virus is causing enormous threats to people’s health and lives, so quantifying the scientific productivity on mental health in times of pandemic is an urgent need, especially to expand the degree of knowledge on mental health problems in regions of low scientific productivity. The aim was to characterize the bibliometric indicators of scientific productivity on mental health during the pandemic in the PubMed Identifier database of the National Library of Medicine in the United States. *Materials and Methods:* A documentary study (bibliometric) of the scientific productivity on mental health in times of pandemic from January 2020 to June 2021 was carried out. The PubMed database was used to abstract the information from the original scientific articles. The data abstracted were: authors, year of publication, journal name, country, and language of publication. *Results:* We identified 47 original articles worldwide, which were published in 29 journals and in three languages (English, Spanish, and German). We observed three groups of countries that published on mental health topics. The first group comprised the largest number of publications, which were multicenter studies (six studies), followed by India (five studies), and Italy (four studies). A second group comprised Bangladesh, China, USA, and Spain, with 3 studies each; and a third group comprised 13 countries (Albania, Saudi Arabia, Argentina, Brazil, South Korea, Denmark, Ecuador, Egypt, Greece, Japan, Jordan, Kuwait, and New Zealand) with one study each. *Conclusions:* Bibliometric indicators of scientific productivity on mental health during the COVID-19 pandemic have ostensibly increased. We verified 47 studies in PubMed, which could serve to improve the understanding and management of COVID-19, as well as serve as a thought-provoking means for other countries and researchers to publish on the state of mental health during and post pandemic.

## 1. Introduction

The new SARS-CoV-2 severe acute respiratory syndrome, also called the COVID-19 pandemic, emerged in Wuhan, China, becoming a worldwide public health emergency, causing enormous threats to people’s health and lives [1,2]. Given its epidemiological and biological characteristics, it turned out to be more contagious than previous pandemics, such as SARS-CoV and MERS-CoV [3].

To minimize the spread of the disease and control the spread of infection, many countries implemented quarantine or physical isolation policies worldwide, including short- to medium-term blockades, voluntary home restriction, cancellation of social and public events, and travel restrictions [4]. This has resulted in one third of the world’s population being blocked with restricted movement to contain the spread of the virus [5].

This situation has reduced social interaction as a result of confinement during the COVID-19 pandemic, further increasing the risk of mental health problems with negative psychological outcomes. The social isolation, lack of family contact, absence of social activities within the home, among other factors, have led to boredom, loneliness, and depression at all stages of life. 

In fact, these events have had a profound impact on the mental health of the world’s population [6,7,8], especially during the pandemic, where people in general tend to experience fear of becoming infected with the virus, consequently producing anxiety, stress, and depression [9].

Recently, several studies have shown that the pandemic has caused spatial distancing, social isolation, and quarantine, bringing with it social, economic, and mental consequences. For example, it has caused depression, frustration, fear, grief, anger, shame, despair, boredom, stress, and panic [10,11,12,13], so that the psychological well-being of people around the world has been adversely affected, especially in low- and middle-income countries [12]. 

These consequences represent threats to both the physical and mental health of the world’s population, so researching the scientific productivity on mental health in times of pandemic is relevant.

In this sense, bibliometrics allows the analysis of scientific production, trends of publications by authors, topics, institutions, countries, regions, among other indicators [14]. As far as it is known, there are few bibliometric studies carried out worldwide [15,16], so quantifying the scientific productivity on mental health in times of pandemic is an urgent need, especially to expand the degree of knowledge on mental health problems in regions of low scientific productivity.

Therefore, the aim of this study was to characterize the bibliometric indicators of scientific productivity on the impacts on mental health during the COVID-19 pandemic in the PubMed database of the U.S. National Library of Medicine. To achieve the aim of the study, we proposed the following questions: how many original studies have been published on the impacts of COVID-19 on mental health from January 2020 to June 2021, and in which scientific journals? This study may provide insight into the global research outlook for scientific progress during the health emergency.

## 2. Materials and Methods

A documentary (bibliometric) study of scientific productivity on the impacts on mental health in times of pandemic was conducted. The research data were extracted from the PubMed database of the United States National Library of Medicine (https://pubmed.ncbi.nlm.nih.gov/, accessed on 25 September 2021). This database was used because it has high coverage of journals in English, in addition to containing citations and summaries of biomedical literature, which facilitates the search of various bibliographic resources of the NLM and is the closest to our object of study (mental health).

### 2.1. Search Strategy and Selection Criteria

The search strategy was applied during the period from January 2020 to June 2021. Studies published in English, Spanish, and German were considered. 

To achieve relevance with this bibliometric review, the articles included the following keywords: (1) mental health, stress, mental state; (2) isolation, quarantine, confinement; (3) COVID-19, pandemic, SARS-CoV-2, coronavirus, virus, disease, and infection.

Initially, all keywords were used together, using the booleans “and” and “or” to sort them. Subsequently, these words were grouped into combinations of two or three, and a new search was performed, such as, for example, mental health and COVID-19 and quarantine. 

The following were considered as indicators of scientific productivity: year of publication, journal name, country, and language of publication. In addition, the observation technique was used to extract bibliometric indicators. The indicators of the scientific articles were recorded on an observation sheet. 

The terms indicated were searched for in the title, abstract, and keywords of the manuscripts. Inclusion criteria were: (i) peer-reviewed articles related to health science areas; (ii) articles on COVID-19; (iii) articles providing all required bibliometric indicators listed; and (iv) published in English, Spanish, and German. In the case of studies that included systematic reviews and meta-analyses, these were excluded from the analysis. 

### 2.2. Data Collection

The procedure for extracting the bibliometric indicators was carried out by two of the researchers in this study (MACB and RGC). Each of the observers recorded the information separately. A third observer collated the records of the first two. This was to certify the process of abstracting the information. In cases where there was no coincidence, this third observer verified each of the indicators and made the pertinent corrections. A general matrix of the studies was then obtained, which made it possible to analyze the bibliometric indicators. 

**Figure 1:**
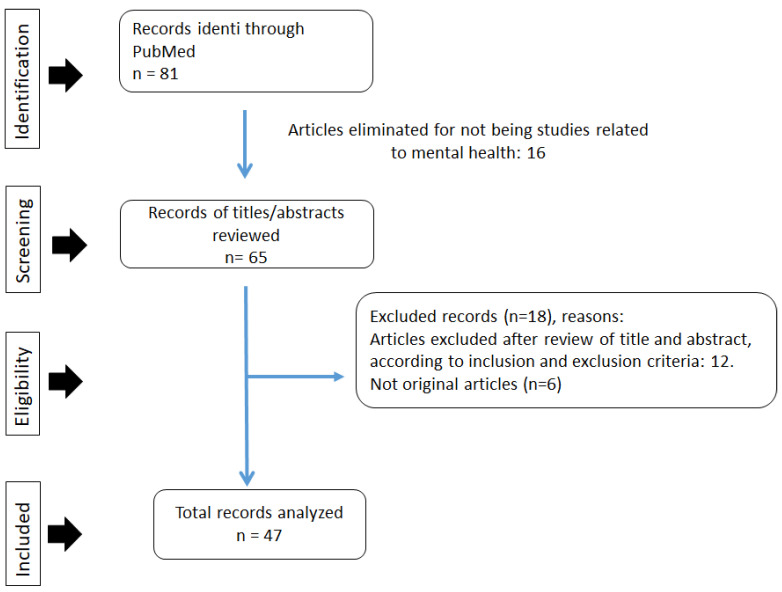
Screening and selection process for the records according to the PRISMA (Preferred Reporting Items for Reviews and Meta-Analyses) flowchart.

We used the PRISMA guidelines, proposed by the researchers Moher, Liberati, Tetzla, and Altman [17], to identify and extract the data for the bibliometric review (Figure 1). A total of 81 scientific articles related to mental health in times of the COVID-19 pandemic were initially identified. Subsequently, 16 studies were eliminated because they were not related to the subject of the study. In the next stage, the titles and abstracts were read and evaluated as to whether they corresponded to the purpose of this research, considering the inclusion criteria, and 12 articles were eliminated. In the third stage, of the 53 eligible studies, bibliographic reviews, systematic reviews, and meta-analyses were excluded. Finally, 47 studies were considered for bibliometric research.

### 2.3. Data Analysis

The data collected from the bibliometric matrix were used to organize the results. These were carried out in Microsoft Excel spreadsheets. Tables and graphs were prepared. Descriptive statistical analyses, such as frequency, range, and percentage (%), were considered to quantify the studies.

## 3. Results

The bibliometric indicators that characterize the studies carried out during 2020 and 2021 related to mental health during the COVID-19 pandemic can be seen in Table 1. A total of 45 studies published in English, and one each in Spanish and German were identified. These total 47 studies. The largest number of publications came from Asia and Europe, with 16 and 17 studies; however, the other continents have reflected between one to three investigations, except for a multicenter study that covered samples from six countries (six studies).

Table 2 shows the list of journals that have published original studies related to mental health during the COVID-19 pandemic. Twenty-nine scientific journals have been identified; PloS ONE was the journal that published the most (11) studies (23.4%) with mental health themes, followed by the Journal of Affective Disorders, with 3 studies (6.4%). Then, six journals have published two studies each, and 21 journals have reported only one study each. Overall, of the seven topics identified, mental well-being was the most researched, totaling 18 publications, followed by: (a) anxiety, depression, and personal stress, and (f) burdens and correlates, and psychological effects, with five publications each. The other topics ranged from one to two studies.

**Figure 2:**
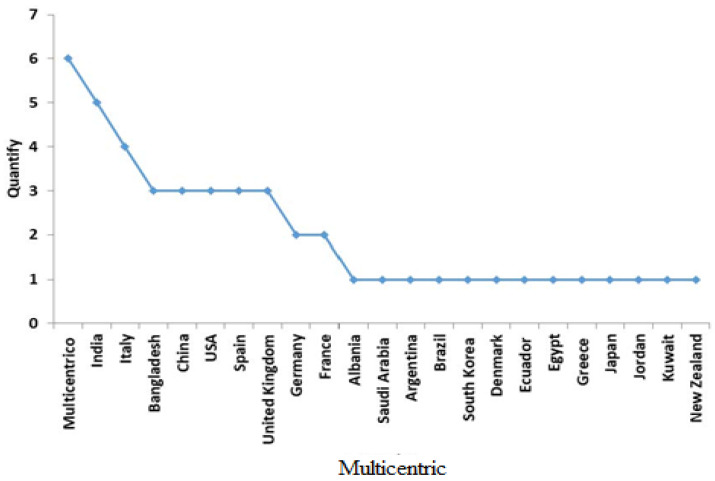
Number of contributions of original articles published on mental health by country during the pandemic (COVID-19).

The number of articles categorized by country is shown in Figure 2. The multicenter study was carried out in six countries, followed by India, which published five studies, Italy with four studies, and Bangladesh, China, USA, and Spain, with three studies each. Subsequently, the other countries published one original article each.

## 4. Discussion

The aim of this study was to characterize the bibliometric indicators of scientific productivity on mental health during the COVID-19 pandemic. In general, 47 articles were identified worldwide, which were published in 29 journals and in three languages (English, Spanish, and German); according to the number of publications, we observed three groups: a first group formed the largest number of publications, including those made up of multicenter studies (with six studies), followed by India (five studies), and Italy (four studies). A second group comprised Bangladesh, China, USA, and Spain, with 3 studies each, and a third group comprised 13 countries (Albania, Saudi Arabia, Argentina, Brazil, South Korea, Denmark, Ecuador, Egypt, Greece, Japan, Jordan, Kuwait, and New Zealand), with one study each.

In fact, these findings are consistent with the study by Gul et al. [18], where they highlight that the countries that produced the most publications worldwide were the USA (North America), Italy (Europe), China (Asia), and the UK (Europe). This evidence highlights the great interest in studying mental health worldwide as a result of the presence of COVID-19 in the years 2020 and 2021. 

For example, Maalouf et al. [19] highlight that, according to the Scopus database up to August 2020, there were more than 43,000 publications on COVID-19, including articles, letters, editorials, notes, reviews, and case reports. On the other hand, in the PubMed database, we found in 2020, 90,659 publications and in 2021 until October 13, 107,000 publications.

This evidence reflects the great progress in scientific publications on various topics and in various publication formats, as it is now widely known that the pandemic has produced a negative impact on mental health worldwide, so the evidence published to date can strengthen the body of knowledge [19] among researchers and health institutions.

Regarding the journals, it was found that PloS ONE had the highest proportion of publications; this is a multidisciplinary journal that corresponds to quartile 1 and is from the United States. The journal with the second highest number of publications was the Journal of Affective Disorders, which is a journal from the Netherlands, and relates to the area of clinical psychology, also located in quartile 1. In addition, we identified the journal BMC Psychiatry (United Kingdom), in which two studies were published, in the areas of psychiatry and mental health.

Overall, a total of 28 countries contributed to the research evidence on mental health problems during the global health emergency during the last 18 months. This research is relevant, since, with the information provided, these countries can generate public policies and intervention programs in their countries to prevent mental health problems in their respective realities [16], while countries that have contributed little or nothing to date should rely on the information evidenced by these reports.

It is widely known that the global COVID-19 pandemic has had a negative impact on mental health worldwide, so it is necessary to strengthen the body of knowledge on mental health in relation to the pandemic [19], as this is the only way to learn more about COVID-19 and, consequently, to counteract outbreaks with appropriate health measures.

The most researched topics were anxiety, depression, and personal stress on the one hand, and on the other hand, burdens and correlates, and psychological effects, with five publications each. 

This is evidence that the COVID-19 pandemic in this period has caused an increase in fear, anxiety, stress, and depression among the general population [20]. Therefore, this reflects the impacts that COVID-19 and its effects on people’s well-being can have, due to its ability to produce a large-scale mental health crisis [21].

In essence, this study presents some strengths, since it is one of the first bibliometric studies describing scientific productivity in the field of mental health. In addition, it can serve as a baseline for future comparisons, and the results described can assist mental health professionals and public policy makers in using this evidence to develop alternatives and roadmaps for implementing clinical interventions, such as telepsychiatry [22].

In addition, this study provided insights into mental health related to COVID-19 outbreaks from a bibliometric perspective. This information can be used by health science professionals to provide care to vulnerable groups, for example, by promoting informative and preventive talks, as well as by producing leaflets and brochures for the general population.

The study has some limitations, since only one database was used, and we limited ourselves to identifying original studies related to mental health in general, so future bibliometric studies should focus on specific age groups, countries with higher numbers of cases of contagion, and outbreaks, among other indicators. This information can help to reduce current research gaps and even to generate future research ideas in mental health.

## 5. Conclusions

This study concludes that bibliometric indicators of scientific productivity on mental health during the COVID-19 pandemic have increased ostensibly. We reviewed 47 studies in PubMed, which could serve as a baseline for improving the understanding and management of COVID-19. In addition, we identified 18 journals that published related topics on mental well-being. These results may serve as a thought-provoking means for other researchers to develop studies in low- and middle-income countries and to publish on the state of mental health during and after a pandemic.

## Figures and Tables

**Table 1:** Characteristics of the bibliometric indicators used.

Indicators	f	%
Publication language		
English	45	95.74
Spanish	1	2.13
German	1	2.13
Total	47	100
Continent		
Asia	16	34.04
Europe	17	36.17
Latin America	3	6.38
North America	3	6.38
Central America		0.00
Africa	1	2.13
Oceania	1	2.13
Multicenter	6	12.77
Total	47	100

**Table 2:** Health science journals indexed in PubMed that published original articles on mental health during the pandemic.

N°	Journals	f	%	Topics Identified						
				a	b	c	d	e	f	g
1	*PLoS ONE*	11	23.40	x	x				x	x
2	*Journal of Affective Disorders*	3	6.38		x		x		x	
3	*BMC Psychiatry*	2	4.26	x						
4	*Community Mental Health Journal*	2	4.26	x						
5	*Eastern Mediterranean Health Journal*	2	4.26		x					
6	*International Journal of Environmental Research and Public Health*	2	4.26		x				x	
7	*Nature research*	2	4.26			x				
8	*Psychiatry Research*	2	4.26		x				x	
9	*Anxiety, Stress, & Coping: An International Journal*	1	2.13		x					
10	*Archives of Psychiatric Nursing*	1	2.13	x						
11	*Archives of Women’s Mental Health*	1	2.13			x				
12	*BMC Pregnancy and Childbirth*	1	2.13		x					
13	*BMC Public Health*	1	2.13		x					
14	*Comprehensive Psychiatry*	1	2.13					x		
15	*Drug and Alcohol Dependence*	1	2.13		x					
16	*European Psychiatry*	1	2.13		x					
17	*Health & Place*	1	2.13		x					
18	*International Journal of Social Psychiatry*	1	2.13	x						
19	*Journal of Biomedical Informatics*	1	2.13		x					
20	*Journal of Clinical Psychology*	1	2.13						x	
21	*Journal of Epidemiology and Community Health*	1	2.13		x					
22	*Journal of Medical Internet Research*	1	2.13		x					
23	*Journal of Psychiatric and Mental Health Nursing*	1	2.13		x					
24	*Nutrients*	1	2.13				x			
25	*Originalarbeit*	1	2.13					x		
26	*Progress in Neuro-Psychopharmacology & Biological Psychiatry*	1	2.13		x					
27	*Revista Española de Salud Pública*	1	2.13				x			
28	*Scandinavian Journal of Public Health*	1	2.13		x					
29	*Translational Behavioral Medicine*	1	2.13		x					

Legend: a: Personal anxiety, depression, and stress; b: Mental well-being; c: Mood; d: Psychological distress; e: Stress levels; f: Burden, correlates, and psychological effects; g: Emotional stimuli.
